# Antenatal depressive symptoms as a predictor of deterioration in perceived social support across the perinatal period: a four-wave cohort study in Turkey

**DOI:** 10.1017/S0033291716002865

**Published:** 2016-11-22

**Authors:** V. Senturk, M. Abas, M. Dewey, O. Berksun, R. Stewart

**Affiliations:** 1 Department of Psychiatry, Ankara University Faculty of Medicine, Ankara, Turkey; 2 King's College London, Institute of Psychiatry, London, UK

**Keywords:** Cohort studies, family structure, perinatal depression, social support

## Abstract

**Background:**

In a perinatal cohort of women in urban and rural Turkey, we investigated associations between antenatal depressive symptoms and subsequent changes in perceived quality of key family relationships.

**Method:**

Of 730 women recruited in their third trimester (94.6% participation), 578 (79.2%) were reassessed at a mean of 4.1 (s.d. = 3.3) months after childbirth, 488 (66.8%) were reassessed at 13.7 (s.d. = 2.9) months, and 448 (61.4%) at 20.8 (s.d. = 2.7) months. At all four examinations, self-reported quality of relationship with the husband, mother and mother-in-law was ascertained using the Close Persons Questionnaire with respect to emotional support, practical support and negative aspects of the relationship. Antenatal depressive symptoms were defined using the Edinburgh Postnatal Depression Scale. A range of covariates in mixed models was considered including age, education, number of children, family structure, physical health, past emotional problems and stressful life events.

**Results:**

Key findings were as follows: (i) reported emotional and practical support from all three relationships declined over time in the cohort overall; (ii) reported emotional support from the husband, and emotional and practical support from the mother-in-law, declined more strongly in women with antenatal depressive symptoms; (iii) associations between depressive symptoms and worsening spouse relationship were more pronounced in traditional compared with nuclear families.

**Conclusions:**

Antenatal depressive symptoms predicted marked decline in the quality of key relationships over the postnatal period. This may account for some of the contemporaneous associations between depression and worse social support, and may compound the risk of perinatal depression in subsequent pregnancies.

## Introduction

Depression is common in women, particularly during the perinatal period. A recent review by Howard *et al*. (2014) concluded that the point prevalence of combined major and minor depression ranged from 8.5–11.0% during pregnancy and from 13.0–19.2% in the first 3 months postpartum. The family environment is clearly important and worse interpersonal relationships have often been found to be associated with perinatal depression in both high- and low-income settings. However, most research in this area has been cross-sectional and the direction of causal relationships between depression and relationship quality has not been fully evaluated. As well as adverse effects of poor interpersonal relationships on risk of depression, it is also possible that depression may exert an adverse effect on the quality of relationships – an issue which has important prognostic implications (Prince *et al.* 2007), particularly in societies with close-knit family structures, but one which has received very little investigation.

In order to address this question, we analysed data from a prospective study of perinatal mental disorder in Turkey which had recruited women in the third trimester of pregnancy and followed them over three subsequent examinations to 20 months postpartum. The study was designed to investigate social support as an exposure, focusing on three key relationships of high importance for women in Middle Eastern settings: that with the husband, the mother and the mother-in-law. We had previously found that lower reported quality of these relationships (particularly with the spouse and mother-in-law) was associated contemporaneously with antenatal depression (Senturk *et al.* 2011); however, the role of the same relationships in predicting postnatal depression was less evident in this cohort at follow-up (Cankorur Senturk *et al.* 2015). We therefore considered the opposite direction of causality (i.e. depression causing worse relationship quality, rather than worse relationship quality causing depression) and sought to test a hypothesized association between antenatal depression and decline in these levels of social support over the perinatal period. We had also found that the association between lower-quality spouse relationship and antenatal depression was stronger in traditional compared with nuclear family settings (Senturk *et al*. 2011), and therefore investigated family structure as a potential modifier in the prospective analysis described here. Turkey is almost unique as a nation in the length of time over which modern Western (‘nuclear’) and traditional Middle Eastern (‘extended’) family structures have co-existed and therefore a promising environment in which to investigate their role as a modifier. Comparisons between different family structures are important for women's mental health because of the rapid ‘Westernization’ of families occurring in many settings around the world.

## Method

### Study design, setting and recruitment sites

The study was carried out in Ankara, central Turkey. Baseline and first follow-up examinations have been previously described (Senturk *et al.* 2011; Cankorur Senturk *et al.* 2015). In summary, baseline samples were drawn from 20 urban and semi-rural antenatal clinics, where all women attending routine third trimester antenatal examinations were approached (December 2007 to August 2008). Attempts were then made to re-contact and interview previous participants as close as possible to 2, 12 and 18 months after their childbirth. The study received approval by ethics committees at Ankara University Faculty of Medicine and King's College London. After complete description of the study to the subjects, written informed consent was obtained at all examinations.

### Samples and follow-up

Of 730 participants assessed in their third trimester (94.6% participation rate), 578 (79.2%) were reassessed at a mean 4.1 (s.d. = 3.3) months after childbirth, 488 (66.8%) were reassessed at 13.7 (s.d. = 2.9) months, and 448 (61.3%) at 20.8 (s.d. = 2.7) months. The main reason for loss to follow-up (17%) between the first two examinations was migration of families due to local re-allocation of housing around that time and consequent loss of contact; 37 (5%) refused.

### Measurements

#### Sociodemographic information

Age, years of education, marital status, current physical health, previous mental health life stressors, number of children, index child health and family structure information were gathered at each examination. Because almost all (97.8%) participants were married and cohabiting with their husband, this was not considered as a covariate. General physical health was self-categorized into five groups: very good, good, average, poor and very poor. Previous mental health was categorized as a binary variable on the basis of any self-reported previous diagnosis of depression, other psychiatric illness or emotional problems in the past. Participants were asked about the presence of the following life stressors/events within the last 12 months (Norbeck & Tilden, 1983): being in debt, hunger from lack of food, recent separation, problems with friends, recent illness/injury, domestic violence, serious illness in a relative, death of a close family member, death of another relative, problems with a job, problems with money, problems with the justice system, any robbery. Finally, family structure was ascertained and classified. A nuclear family structure was defined as a wife and husband living alone or with their children in the same household, whereas a traditional/extended family structure was defined if another adult was living with the married couple in the same household. In Turkish society this would nearly always be the participant's mother-in-law or father-in-law.

#### Depressive symptoms

The Edinburgh Postnatal Depression Scale (EPDS; Cox *et al*. 1987) was chosen for this study as one of the most widely used screening instruments for perinatal depression internationally (Gaynes *et al.* 2005) and the most commonly used in previous Turkish research. It was administered in an identical format at all examinations. The maximum score on the EPDS is 30 and a score of 13 or above was used to classify case status for antenatal depressive symptoms, as has been most commonly applied in previous Turkish samples (Engindeniz *et al.* 1996; Aydin *et al.* 2005). Women with depressive symptoms were not formally re-evaluated clinically. However, women with moderate or severe depressive symptoms and wishing treatment were referred to their general practitioner or to a psychiatrist.

#### Quality of relationships and social support

The Close Persons Questionnaire (CPQ; Stansfeld & Marmot, 1992) was administered in identical format at all four examinations and comprised the outcome for these analyses. The CPQ includes a 15-item scale ascertaining participants' perceptions of three types of support from a nominated person nominated: (*a*) confiding/emotional support; (*b*) practical support; and (*c*) negative aspects of close relationships (Stansfeld & Marmot, 1992). In conventional use of the measurement, the participant is asked to nominate the person (or two to four persons) closest to them and the scale is then used to infer an overall picture of social support based on that or those closest relationship(s). The study described here deviated from this protocol and applied/imposed the scale to three relationships anticipated *a priori* to be the most important for Turkish women in their perinatal period: i.e. the husband, the mother and the mother-in-law. Data were coded as missing on these sections if this information could not be obtained (e.g. if the mother or mother-in-law was deceased). In other respects, application of the scale was standard.

### Statistical analyses

Changes in social support measures were initially displayed by cross-tabulating mean scores by examination point in participants with and without case-level depressive symptoms (referred to hereafter as depression for brevity) at baseline. SPSS 19 statistical software (USA) was used for analyses. Mixed models were then applied to these changes and were constructed as follows: the score for the individual social support measure was entered as the dependent variable with time (continuous variable), baseline depression (binary variable) and an interaction term between the two as the principal independent variables. Time was measured in months but was analysed in year units to give coefficients in a sensible range. Of the coefficients generated, that for depression represented the difference in intercept between the two groups, the coefficient for time represented the overall change in the social support measure for participants without baseline depression, and the coefficient for the interaction term represented the difference in this slope between participants with and without baseline depression. Potential confounding factors were added in blocks with the following models: model 1 adjusted for age; model 2 adjusted for age plus education, number of children, family structure, physical health, and any past emotional problems; model 3 adjusted for all above variables plus adverse life events at baseline. Nuclear *v.* traditional/extended family structure was additionally investigated as an effect modifier. The following sensitivity analyses were carried out: (i) using mixed models which replaced depression at baseline with depression at each examination point modelling relationship quality trajectories accordingly; (ii) using mixed models including postnatal depression as a covariate.

## Results

### Sample characteristics at baseline

Sample characteristics are described in Table 1. Participants lost to follow-up had more children and reported more negative aspects of key relationships at baseline, but those followed or not were otherwise similar. In particular, there was no difference in antenatal depression prevalence. From supplementary questionnaire items, the majority (88.1%) reported a ‘good’ or ‘very good’ relationship with their husband, and very few (2.5%) reported any physical abuse since giving birth. Almost all of the participants (99.1%) gave birth at health facilities and 64.2% had a natural delivery. All participants gave birth to a live baby (50.5% male). Two participants gave birth to twins and one baby died after birth; 97.1% of babies were vaccinated before the follow-up interview.

**Table 1.** Sociodemographic characteristics of the sample at baseline who were followed or not at the fourth examination

	Baseline characteristics								
	All participants (*n* = 730)		Participants present at fourth examination (*n* = 448)		Participants not present at fourth examination (*n* = 282)		Difference between those followed and not followed		
	Mean (s.d.)	*n* (%)	Mean (s.d.)	*n* (%)	Mean (s.d.)	*n* (%)	χ^2^ or *t*	df	*p*
Age, years	25.8 (5.2)		26.1 (5.1)		25.3 (5.3)		6.33	3	0.09
18–22		211 (29.1)		116 (27.0)		95 (32.2)			
23–25		169 (23.3)		93 (21.7)		76 (25.8)			
26–29		173 (23.9)		108 (25.2)		65 (22.0)			
30–44		171 (23.6)		112 (26.1)		59 (20.0)			
Number of children							7.74	2	0.02*
0		386 (53.0)		211 (48.8)		175 (59.1)			
1		231 (31.7)		147 (34.0)		84 (28.4)			
2+		111 (15.2)		74 (17.1)		37 (12.5)			
Education duration; years	8.4 (4.4)		8.3 (5.0)		8.4 (3.6)		1.48	3	0.68
⩽5		232 (33.1)		144 (34.7)		88 (30.8)			
6–8		145 (20.7)		82 (19.8)		63 (22.0)			
9–11		241 (34.4)		139 (33.5)		102 (35.7)			
12+		83 (11.8)		50 (12.0)		33 (11.5)			
Physical health							0.32	2	0.85
Very good		129 (17.8)		75 (17.5)		54 (18.4)			
Good		449 (62.1)		266 (61.9)		183 (62.5)			
Average/bad/very bad		145 (20.1)		89 (20.7)		56 (19.1)			
No life events							2.40	4	0.66
0		320 (49.5)		200 (52.4)		120 (45.5)			
1		172 (26.6)		96 (25.1)		76 (28.8)			
2		87 (13.5)		48 (12.6)		39 (14.8)			
3+		67 (10.4)		38 (10.0)		29 (11.0)			
Emotional problems in the past							0.41	1	0.53
No		361 (51.3)		219 (52.3)		142 (49.8)			
Yes		343 (48.7)		200 (47.7)		143 (50.2)			
Family structure							1.37	1	0.24
Nuclear		478 (65.7)		291 (67.4)		187 (63.2)			
Traditional		250 (34.2)		141 (32.6)		109 (36.8)			
Antenatal depression							0.91	1	0.34
No		489 (66.9)		296 (68.4)		193 (65.0)			
Yes		241 (33.1)		137 (31.6)		104 (35.0)			
Mean social support indices	
From husband	
Emotional	26.1 (5.4)		27.1 (4.6)		26.3 (5.4)		−0.79	695	0.30
Practical	9.6 (2.2)		9.7 (2.1)		9.8 (2.2)		0.1	707	0.84
Negative aspects	10.8 (2.3)		9.9 (2.0)		10.9 (2.4)		1.1	690	0.01*
From mother	
Emotional	24.4 (5.6)		24.9 (5.4)		25.1 (5.3)		0.31	663	0.70
Practical	8.5 (2.9)		9.0 (2.7)		8.9 (2.8)		−0.1	668	0.82
Negative aspects	9.6 (2.2)		9.1 (1.9)		9.8 (2.2)		0.73	653	0.02*
From mother-in-law	
Emotional	18.6 (6.4)		19.3 (6.0)		18.7 (6.4)		−0.54	633	0.59
Practical	6.9 (3.1)		7.7 (2.8)		6.9 (3.1)		−0.79	647	0.08
Negative aspects	9.8 (2.6)		9.2 (1.8)		9.9 (2.7)		0.71	631	0.08

s.d., Standard deviation; df, degrees of freedom.

* *p* < 0.05.

### Associations between antenatal depression and trajectories of social support across the follow-up period

**Fig. 1.**
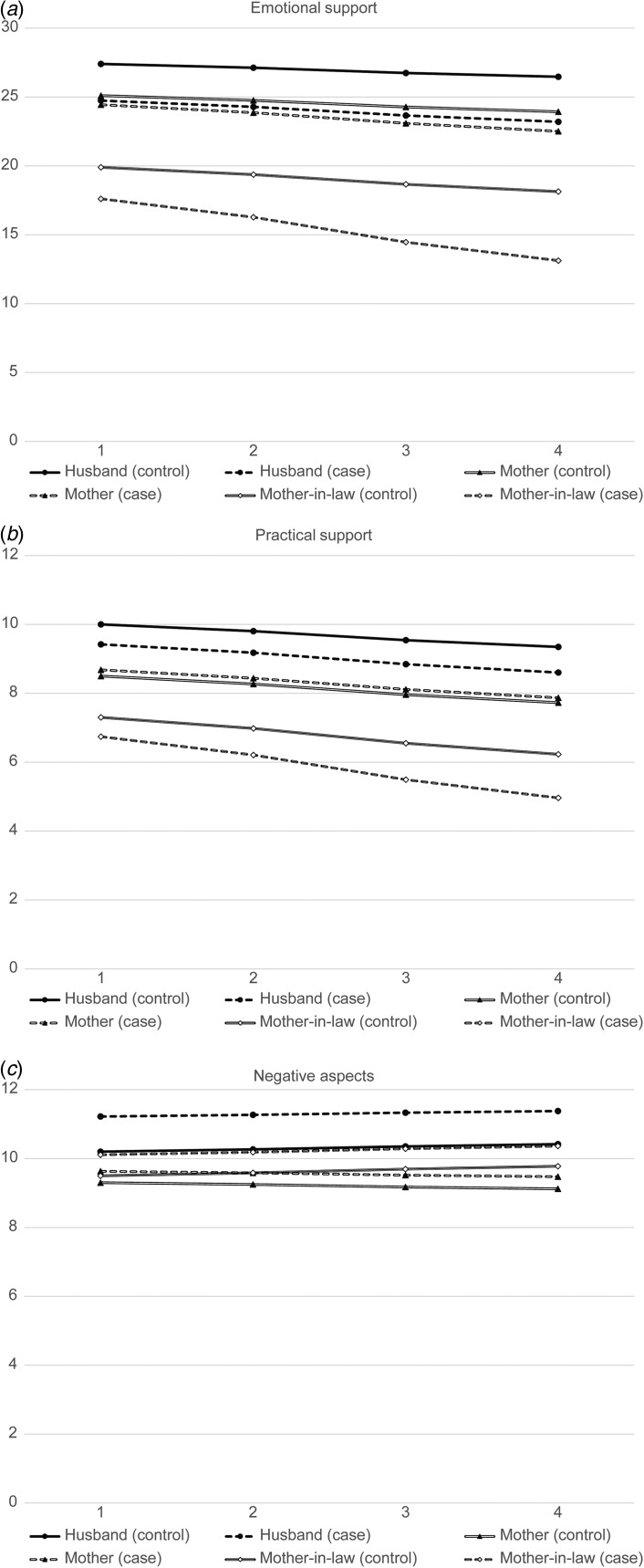
Predicted trajectories of social support across the four examinations in participants with and without depressive symptoms at baseline (termed cases and controls, respectively) for the three outcomes and three relationships of interest. (*a*) Emotional support; (*b*) practical support; (*c*) negative aspects. Derived from fully adjusted coefficients (Table 2, model 3).

Online Supplementary Table S1 summarizes mean scores for the social support measures by exposure group and examination and Table 2 displays output from the mixed models considering the unadjusted and adjusted trends in social support measures and differences between participants with and without baseline (antenatal) depression. Intercept coefficients indicated worse self-rated social support on all three CPQ subscales for relationships with the husband and mother-in-law and on one subscale for the relationship with the mother. These were consistent with cross-sectional analyses of baseline data previously reported (Senturk *et al.* 2011). Time terms for emotional and practical support were negative, indicating worsening scores over the subsequent examinations across the sample as a whole – significant for all social support from mother-in-law and only practical support from husband in all models. From visual inspection of online Supplementary Table S1 there was no consistent pattern across these measures in the timing of the deterioration to indicate non-monotonic patterns of decline. Time terms for negative aspects were not significant for any relationship except mother-in-law. Interaction terms between baseline depression and time were significant for emotional support from the husband, and emotional and practical support from the mother-in-law. The positive value of these coefficients, coupled with the negative coefficients for time, indicated a more rapid deterioration in these measures of social support in women with depressive symptoms at baseline. In fully adjusted models, considering interaction terms as the key output of interest, coefficients remained similar to those in unadjusted models. Predicted trajectories based on model 3 coefficients (and on online Supplementary Table S1 baseline mean scores in those without depression) are visually displayed in Fig. 1 for each of the three outcomes.

**Table 2.** Mixed-model estimations of baseline depression, time and their interaction as predictors of social support over the four examinations^a^

Nature of support (dependent variable) and predictor	B-coefficient (95% CI)			
	Unadjusted	Model 1	Model 2	Model 3
From husband				
Emotional support				
Intercept	−3.68 (−4.49 to −2.87)*	−3.68 (−4.48 to −2.88)*	−3.13 (−3.97 to −2.29)*	−2.66 (−3.50 to −1.82)*
Time	−0.48 (−0.79 to −0.17)*	−0.47 (−0.78 to −0.16)*	−0.47 (−0.79 to −0.16)*	−0.47 (−0.79 to −0.16)*
Interaction	0.64 (0.07 to 1.21)*	0.64 (0.07 to 1.21)*	0.66 (0.09 to 1.23)*	0.65 (0.08 to 1.22)*
Practical support				
Intercept	−0.87 (−1.21 to −0.53)*	−0.88 (−1.22 to −0.54)*	−0.73 (−1.09 to −0.37)*	−0.58 (−0.94 to −0.22)*
Time	−0.33 (−0.49 to −0.18)*	−0.33 (−0.48 to −0.18)*	−0.33 (−0.48 to −0.18)*	−0.33 (−0.48 to −0.18)*
Interaction	0.25 (−0.02 to 0.53)	0.25 (−0.02 to 0.53)	0.25 (−0.03 to 0.52)	0.25 (−0.03 to 0.52)
Negative aspects of the relationship				
Intercept	1.32 (0.96 to 1.68)*	1.36 (0.99 to 1.72)*	1.12 (0.74 to 1.50)*	1.02 (0.63 to 1.40)*
Time	0.11 (−0.07 to 0.29)	0.10 (−0.07 to 0.28)	0.11 (−0.07 to 0.29)	0.11 (−0.07 to 0.29)
Interaction	−0.25 (−0.57 to 0.07)	−0.25 (−0.57 to 0.07)	−0.27 (−0.60 to 0.05)	−0.27 (−0.60 to 0.05)
From mother				
Emotional support				
Intercept	−1.51 (−2.48 to −0.54)*	−1.50 (−2.46 to −0.54)*	−1.11 (−2.09 to −0.13)*	−0.66 (−1.65 to 0.33)
Time	−0.61 (−1.00 to −0.22)*	−0.60 (−0.98 to −0.21)*	−0.59 (−0.98 to −0.21)*	−0.59 (−0.97 to −0.21)*
Interaction	0.63 (−0.08 to 1.34)	0.63 (−0.08 to 1.33)	0.66 (−0.04 to 1.37)	0.65 (−0.05 to 1.35)
Practical support				
Intercept	−0.08 (−0.54 to 0.37)	−0.09 (−0.55 to 0.36)	0.06 (−0.40 to 0.52)	0.18 (−0.29 to 0.66)
Time	−0.40 (−0.59 to −0.22)*	−0.40 (−0.58 to −0.21)*	−0.39 (−0.57 to −0.20)*	−0.39 (−0.57 to −0.20)*
Interaction	0.06 (−0.29 to 0.40)	0.06 (−0.29 to 0.40)	0.06 (−0.29 to 0.40)	0.05 (−0.29 to 0.39)
Negative aspects of the relationship				
Intercept	0.39 (0.04 to 0.75)*	0.40 (0.05 to 0.75)*	0.34 (−0.03 to 0.70)	0.33 (−0.05 to 0.70)
Time	−0.09 (−0.27 to 0.08)	−0.09 (−0.27 to 0.08)	−0.09 (−0.27 to 0.09)	−0.09 (−0.27 to 0.09)
Interaction	−0.10 (−0.42 to 0.22)	−0.11 (−0.43 to 0.22)	−0.12 (−0.44 to 0.21)	−0.12 (−0.44 to 0.21)
From mother-in-law				
Emotional support				
Intercept	−3.27 (−4.35 to −2.18)*	−3.30 (−4.34 to −2.27)*	−2.68 (−3.74 to −1.63)*	−2.29 (−3.36 to −1.23)*
Time	−0.90 (−1.27 to −0.53)*	−0.88 (−1.25 to −0.51)*	−0.89 (−1.26 to −0.52)*	−0.89 (−1.26 to −0.52)*
Interaction	1.47 (0.80 to 2.13)*	1.48 (0.82 to 2.15)*	1.54 (0.88 to 2.20)*	1.54 (0.88 to 2.20)*
Practical support				
Intercept	−0.83 (−1.33 to −0.34)*	−0.88 (−1.36 to −0.40)*	−0.72 (−1.19 to −0.24)*	−0.56 (−1.04 to −0.08)*
Time	−0.56 (−0.74 to −0.37)*	−0.54 (−0.73 to −0.36)*	−0.54 (−0.73 to −0.36)*	−0.54 (−0.73 to −0.36)*
Interaction	0.64 (0.31 to 0.97)*	0.65 (0.31 to 0.98)*	0.66 (0.33 to 0.99)*	0.66 (0.33 to 0.99)*
Negative aspects of the relationship				
Intercept	0.79 (0.34 to 1.23)*	0.76 (0.32 to 1.20)*	0.60 (0.15 to 1.06)*	0.61 (0.15 to 1.08)*
Time	0.11 (−0.11 to 0.33)	0.12 (−0.10 to 0.34)	0.14 (−0.08 to 0.35)	0.14 (−0.08 to 0.36)
Interaction	−0.06 (−0.45 to 0.33)	−0.06 (−0.45 to 0.33)	−0.09 (−0.48 to 0.31)	−0.09 (−0.48 to 0.31)

CI, Confidence interval.

^a^ Model 1: adjusted for age (categorized into four groups: 18–22, 23–25, 26–29, 30+ years). Model 2: model 1 plus education (four groups: <6, 6–8, 9–11, 12+ years), number of children (three groups: 0, 1, 2+), family structure (traditional/nuclear), physical health (three groups: very good, good and average/poor/very poor), past emotional problems (binary variable). Model 3: model 2 plus adverse life events at baseline (four groups: 0, 1, 2, 3+).

* *p* < 0.05.

### Further analyses

The associations between baseline depression and trajectories of social support are further compared between nuclear and traditional family structures in online Supplementary Table S2. The time coefficients were generally similar between the two strata. The depression × time interaction terms predicting emotional and practical support from the husband were substantially stronger in the traditional compared with the nuclear family structure but those for the mother-in-law relationship were similar. Exploratory mixed models were carried out entering depression at each time point as the primary independent variable and are displayed in online Supplementary Table S3. Coefficients were understandably smaller in value because of the new parameters; however, broadly speaking the pattern was similar to Table 2, with negative ‘intercept’ coefficients strongest for the husband and mother-in-law relationships, significant time coefficients for practical support from the husband and all three measures from the mother-in-law, and a significant depression × time interaction term for practical support from the mother-in-law. A final analysis applying postnatal depression as a covariate had negligible effect, with very little change in coefficients from before to after adjustment (online Supplementary Table S4).

## Discussion

In a large cohort of women, changes in self-rated quality of support from the husband, mother and mother-in-law were modelled over four perinatal examinations. Key findings were as follows: (i) self-rated emotional and practical support from all three relationships worsened over time in the cohort overall; (ii) emotional support from the husband, and emotional and practical support from the mother-in-law declined more strongly in women with depressive symptoms at baseline; (iii) the association between depressive symptoms and marked decline in emotional and practical support from the husband was more pronounced in traditional compared with nuclear families.

Lower social support has been reported to be a strong to moderate risk factor for postnatal depression (Rubertsson *et al.* 2005) and a moderate risk factor for antenatal depression (Lancaster *et al.* 2010). However, there has been little or no investigation of the opposite association – i.e. the extent to which depression is a risk factor for deterioration in social support. The findings reported here suggest a reciprocal relationship, with an overall decline in several aspects of social support in the cohort as a whole over the postnatal period which was more marked in women with antenatal depression at baseline. The fact that associations with depression were most marked for reported emotional support rather than other aspects, and most marked for reported support from the husband and mother-in-law rather than the mother, is consistent with other analyses in this cohort. Specifically, in cross-sectional analyses at baseline, lower-quality reported relationships with key family members were strongly associated with third trimester depressive symptoms, particularly relationships with the husband and mother-in-law, and stronger in traditional compared with nuclear family settings (Senturk *et al.* 2011). In analyses of data from the first two examinations, the incidence and persistence of depressive symptoms were also predicted by lower baseline reported emotional support from the mother-in-law and the husband, respectively (Cankorur Senturk *et al.* 2015). No predictive associations were found for practical support or negative aspects of the relationships, which might reflect different psychometric properties between the subscales, but might alternatively reflect more salient features of the relationship quality for participants. Antenatal depressive symptoms were associated with a marked decline in emotional support from the husband and with both emotional and practical support from the mother-in-law; however, the mutual independence of these associations was not investigated, and they are likely to be related constructs.

As far as we are aware, ours is the first study investigating depression as a predictor of decline in social support over the perinatal period. However, a longitudinal study of a Canadian community sample found reciprocal relationships between major depression and low social support: the strongest and most robust findings were for low support as a risk factor for depression, but depression also predicted the emergence of low ‘affection social support’ (Patten *et al.* 2010). A study in Finland following people with major depressive disorder over an 18-month period found an improvement in subjective support associated with clinical recovery but no improvement in objective support, and both outcomes deteriorated in persistent cases (Leskelä *et al.* 2008). Studies investigating so called ‘scar effects’ of depression (i.e. persisting negative psychological change after symptomatic resolution) have tended not to find evidence for this (Beevers *et al.* 2007). Instead, the concept of ‘erosive effects’ (depression-induced changes in perceptions of social support leading to counterproductive behaviours such as the seeking of reassurance and/or negative feedback) has been suggested as a more plausible hypothesis (Joiner, 2000).

The stronger associations between depressive symptoms and reported support from the mother-in-law rather than the mother, observed both in this and previous analyses in this cohort (Senturk *et al*. 2011; Cankorur Senturk *et al*. 2015), may reflect the relative importance of the former relationship for married women in Turkish society. However, another potential explanation is that the perceived relationship with the mother-in-law was a marker of the quality of the marriage, since a worse-quality relationship of this sort might well place pressures on the spousal relationship, and might have been more acceptable for participants to report at interview. Finally, there might have been a reluctance on the part of participants to report problems with parental relationships, particularly emotional relationships. Support from family members has been found to be an important buffer against depression in women from other low- and middle-income settings (Broadhead *et al.* 2001) and some research in Islamic nation settings has suggested both high prevalence of perinatal disorder and a potentially harmful role of disruptions to traditional family structures (Rahman *et al.* 2003). If maternal depression has a deleterious impact on relationships with the spouse and mother-in-law, this could have important longer-term consequences in terms of recovery and recurrence – issues which need further investigation, particularly in the traditional extended family settings where large numbers of women continue to live.

A traditional family structure appeared to increase the impact of depression on the spousal relationship but did not apparently modify the impact on the mother-in-law relationship. It is important to note that no differences were found in this cohort between traditional and nuclear family settings in the prevalence of antenatal depressive symptoms (Senturk *et al.* 2011) or in incidence/maintenance of depressive symptoms at the first postnatal examination (Cankorur Senturk *et al.* 2015), and there was also no apparent influence of the family setting on changes of relationship quality across the whole sample. This is consistent with the lack of association found between nuclear family settings and postnatal depression in Bangladesh (Gausia *et al.* 2009), although extended families were protective in a Pakistan study of antenatal and postnatal depression, particularly support from family members with routine child-care and the presence of the infant's grandmother (Rahman *et al*. 2003).

Strengths of this study include its prospective design and the particular features of the setting, as previously mentioned, generating a large and heterogeneous sample of women in different family structures. Follow-up rates were reasonable and refusal rates low, reducing the risk of selection bias. In addition, baseline characteristics did not differ substantially between those present or not at follow-up – most importantly, attrition was not predicted by depressive symptoms or by the most salient social support measures (i.e. those concerning the husband and mother-in-law) at baseline. Sensitivity analyses did not indicate that postnatal depression was an important covariate (i.e. mediating factor).

A comprehensive range of covariates was taken into account, reducing the likelihood of confounding, although this cannot be ruled out entirely. For example, there was no specific information collected on participants' own upbringing, family structure or prior family relationships. In addition, personality traits were not measured, and previous physical and mental disorders were ascertained from self-report rather than records access. It should also be considered that the mental health of the husband might have an effect on an association between maternal mental health and social support during the perinatal period (Paulson & Bazemore, 2010), but unfortunately we were not able to evaluate this as a potential confounder or modifier.

As well as unmeasured confounding factors, there are additional features of the study which should be considered when interpreting findings. Considering the depression classification, the EPDS has been widely used in international research; however, it should be borne in mind that it is a screening instrument, measuring number of depressive symptoms and not seeking to define specific depression syndromes or to apply diagnostic criteria. Considering family structure, while it is our belief that the different structures characterized represent heterogeneity in experience, it is possible that there are societal norms and expectations in Turkey which transcend these structural differences (for example, pertaining to the importance of the mother-in-law relationship even where there is no co-residence). Also, it is important to bear in mind that the nuclear and traditional family structures investigated in this study have coexisted over a long period in Turkey. Findings therefore may not generalize to other societies where nuclear families are a relatively recent phenomenon and potentially less supported and/or more stigmatized. An important consideration is that it is not possible to infer with certainty whether the more marked decline in reported support represented a consequence of the antenatal depressive episode or whether both reflected ongoing or emerging poor relationships preceding the depression. Clarification of this issue would require research over a much longer period, ideally from a point preceding first pregnancies or possibly even the marriage itself. Regression to the mean is not a valid explanation, since levels of support were already relatively low at baseline in participants with depression and this process would have obscured rather than marked associations with subsequent decline. Finally, although we believe that the study achieved a close-to-naturalistic follow-up, there was an ethical duty on researchers to encourage general practitioner contact for women found to have severely distressing symptoms, as would have been the case for those attending any perinatal service, so it is possible that the sample received more intervention than would have occurred in normal circumstances.

Our study's focus was on the relationship between antenatal depressive symptoms and subsequent trajectories in self-reported relationship quality. There are a number of causal pathway factors, as well as potential effect modifiers, occurring during the period of outcome measurement which were beyond the scope of this study to investigate. For example, the role of experiences around and following childbirth was not considered, nor was the infant temperament or maternal–infant relationship, although it is reasonable to assume that these might at least modify the association of interest (e.g. antenatal depression might have a stronger effect on declining relationship quality if there are additional pressures arising from a more traumatic subsequent birth and/or difficulties with mother–infant bonding). The persistence or not of depressive symptoms into the postnatal period is also likely to be important and is supported by supplementary analyses reported in online Supplementary Table S3. These indicate that case-level depression at all points in the study was associated with lower contemporaneous self-rated support from the husband and mother-in-law, and a more marked decline in relationship quality with the mother-in-law.

The importance of this study is in its demonstration of a potentially deleterious influence of depression on levels of social support from close family members. This suggests that at least some of the well-recognized contemporaneous associations between depression and lower social support are due to an effect of the former on the latter, rather than the more normally assumed role of low social support as a risk factor; the relationship may therefore be bidirectional. However, beyond this contribution towards clarifying direction of cause and effect in a research context, there are potentially important clinical implications. In particular, there is a need to establish the extent to which adverse consequences of one episode of depression may create an environment which increases risk of prolonged or recurrent episodes later on, whether associated with subsequent pregnancies or more generally. It is also important to evaluate the impact of depression and declining social support both together and independently of offspring development. There may be scope for interventions specifically focusing on improving relationship quality in the perinatal period for women with depressive symptoms, and evaluative studies are warranted to ascertain not only whether these are effective in themselves, but also whether they prevent future episodes of depressive disorder.

## Supplementary material

For supplementary material accompanying this paper visit http://dx.doi.org/10.1017/S0033291716002865. click here to view supplementary material

## References

[ref1] Aydin N, Inandi T, Karabulut N (2005). Depression and associated factors among women within their first postnatal year in Erzurum province in eastern Turkey. Women and Health 41, 1–12. 10.1300/J013v41n02_01 16219584

[ref2] Beevers CG, Rohde P, Stice E, Nolen-Hoeksema S (2007). Recovery from major depressive disorder among female adolescents: a prospective test of the scar hypothesis. Journal of Consulting and Clinical Psychology 75, 888–900. 18085906 10.1037/0022-006X.75.6.888

[ref3] Broadhead J, Abas M, Khumalo Sakutukwa G, Chigwanda M, Garura E (2001). Social support and life events as risk factors for depression amongst women in an urban setting in Zimbabwe. Social Psychiatry and Psychiatric Epidemiology 36, 115–122. 11465782 10.1007/s001270050299

[ref4] Cankorur Senturk V, Abas M, Berksun O, Stewart R (2015). Social support and the incidence and persistence of depression between antenatal and postnatal examinations in Turkey, a cohort study. BMJ Open 5, e006456. 10.1136/bmjopen-2014-006456 PMC4390689 25833665

[ref5] Cox JL, Holden JM, Sagovsky R (1987). Detection of postnatal depression: development of the 10-item Edinburgh Postnatal Depression Scale. British Journal of Psychiatry 150, 782–786. 3651732 10.1192/bjp.150.6.782

[ref7] Engindeniz AN, Kuey L, Kultur S (1996). Edinburgh dogum sonrası depresyon olcegi Turkce formu gecerlilik ve guvenilirlik calısması In Bahar Sempozyumları 1 Kitabı, pp. 51–52. Psikiyatri Dernegi Yayınları: Ankara.

[ref8] Gausia K, Fisher C, Ali M, Oosthuizen J (2009). Magnitude and contributory factors of postnatal depression: a community-based cohort study from a rural subdistrict of Bangladesh. Psychological Medicine 39, 999–1007. 18812008 10.1017/S0033291708004455

[ref9] Gayness BN, Gavin NI, Meltzer-Brody S, Lohr KN, Swinson T, Gartlehner G, Brody S, Miller WC (2005). Perinatal depression: prevalence, screening accuracy, and screening outcomes. *Evidence Report/Technology Assessment (Summary)*, no. 119, 1–8. 10.1037/e439372005-001 PMC4780910 15760246

[ref10] Howard LM, Molyneaux E, Dennis CL, Rochat T, Stein A, Milgrom J (2014). Non-psychotic mental disorders in the perinatal period. Lancet 384, 1775–1788. 25455248 10.1016/S0140-6736(14)61276-9

[ref11] Joiner TE (2000). Depression's vicious scree: self-propagating and erosive processes in depression chronicity. Clinical Psychology: Science and Practice 150, 720–727.

[ref12] Lancaster CA, Gold KJ, Flynn HA, Yoo H, Marcus SM, Davis MM (2010). Risk factors for depressive symptoms during pregnancy: a systematic review. American Journal of Obstetricians and Gynaecology 202, 5–14. 10.1016/j.ajog.2009.09.007 PMC2919747 20096252

[ref13] Leskelä U, Melartin T, Rytsälä H, Sokero P, Lestelä-Mielonen P, Isometsä E (2008). The influence of major depressive disorder on objective and subjective social support: a prospective study. Journal of Nervous and Mental Disease 196, 876–883. 19077854 10.1097/NMD.0b013e31818ec6cf

[ref14] Norbeck JS, Tilden VP (1983). Life stress, social support, and emotional disequilibrium in complications of pregnancy: a prospective, multivariate study. Journal of Health and Social Behavior 24, 30–46. 6853997

[ref15] Patten SB, Williams JWA, Lavorato DH, Bulloch AGM (2010). Reciprocal effects of social support in major depression epidemiology. Clinical Practice and Epidemiology in Mental Health 6, 126–131. 21253020 10.2174/1745017901006010126 PMC3023950

[ref16] Paulson JF, Bazemore SD (2010). Prenatal and postpartum depression in fathers and its association with maternal depression. JAMA 303, 1961–1969. 20483973 10.1001/jama.2010.605

[ref17] Prince M, Patel V, Saxena S, Maj M, Maselko J, Phillips MR, Rahman A (2007). No health without mental health. Lancet 370, 859–877. 17804063 10.1016/S0140-6736(07)61238-0

[ref18] Rahman A, Iqbal Z, Harrington R (2003). Life events, social support and depression in childbirth: perspectives from a rural community in the developing world. Psychological Medicine 33, 1161–1167. 14580070 10.1017/s0033291703008286

[ref20] Rubertsson C, Wickberg B, Gustavsson P, Rådestad I (2005). Depressive symptoms in early pregnancy, two months and one year postpartum-prevalence and psychosocial risk factors in a national Swedish sample. Archives of Women's Mental Health 8, 97–104. 10.1007/s00737-005-0078-8 15883652

[ref21] Senturk V, Abas M, Berksun O, Stewart R (2011). Social support and antenatal depression in extended and nuclear family environments in Turkey: a cross-sectional survey. BMC Psychiatry 24, 11–48. 10.1186/1471-244X-11-48 PMC3073894 21435209

[ref22] Stansfeld S, Marmot M (1992). Deriving a survey measure of social support: the reliability and validity of the Close Persons Questionnaire. Social Science and Medicine 35, 1027–1035. 1411697 10.1016/0277-9536(92)90242-i

