# Supplementary material for: Antenatal depressive symptoms as a predictor of deterioration in perceived social support across the perinatal period: a four-wave cohort study in Turkey
Source: Psychol Med. 2016 Nov 22;47(4):766–75. doi: 10.1017/S0033291716002865 (PMC5426317; doi:10.1017/S0033291716002865)
Supplement: Supplementary file 1 [file S0033291716002865sup001.doc]

| **Supplementary Table S1.** *Social support scores by examination and baseline depression status* | | | | | | | | |
| --- | --- | --- | --- | --- | --- | --- | --- | --- |
| Social support scale (Close Persons Questionnaire) | Mean social support scores | | | | | | | |
|  | No depression at baseline (n=492) | | | | Depression at baseline (n=238) | | | |
|  | Exam 1 | Exam 2 | Exam 3 | Exam 4 | Exam 1 | Exam 2 | Exam 3 | Exam 4 |
| *From husband* |  |  |  |  |  |  |  |  |
| Emotional | 27.4 | 26.6 | 26.6 | 26.3 | 23.4 | 23.4 | 23.3 | 23.9 |
| Practical | 10.0 | 9.8 | 9.5 | 9.2 | 8.9 | 9.3 | 8.6 | 8.1 |
| Negative aspects | 10.2 | 9.4 | 10.3 | 10.3 | 11.8 | 10.7 | 11.3 | 11.4 |
| *From mother* |  |  |  |  |  |  |  |  |
| Emotional | 25.1 | 23.9 | 22.5 | 24.1 | 23.2 | 22.8 | 21.3 | 23.6 |
| Practical | 8.5 | 8.5 | 7.6 | 7.7 | 8.4 | 8.4 | 7.0 | 8.1 |
| Negative aspects | 9.3 | 8.7 | 9.1 | 9.0 | 10.0 | 8.9 | 9.3 | 9.6 |
| *From mother in law* |  |  |  |  |  |  |  |  |
| Emotional | 19.9 | 19.2 | 17.6 | 18.3 | 16.0 | 17.2 | 15.7 | 17.2 |
| Practical | 7.3 | 7.6 | 6.1 | 6.3 | 6.0 | 7.3 | 5.8 | 6.4 |
| Negative aspects | 9.5 | 8.4 | 8.9 | 9.6 | 10.3 | 9.4 | 10.2 | 10.1 |

| **Supplementary Table S2.** *Mixed model estimations of baseline depression, time and their interaction as predictors of social support over the four examinations, stratified by nuclear and traditional family structure* | | | |
| --- | --- | --- | --- |
| Nature of support (dependent variable) and predictor | B-coefficients (95% CI) from fully adjusted model (Model 3 from Table 2) | | |
|  | Full sample | Nuclear family | Traditional family |
| **From husband** |  |  |  |
| *Emotional support* | |  |  |
| Intercept | -2.66 (-3.50, -1.82)* | -1.93 (-2.97, -0.90)* | -4.04 (-5.56, -2.52)* |
| Time | -0.47 (-0.79, -0.16)* | -0.19 (-0.58, 0.19) | -1.07 (-1.60, -0.54)* |
| Interaction | 0.65 (0.08, 1.22)* | 0.20 (-0.51, 0.90) | 1.60 (0.64, 2.56)* |
| *Practical support* | |  |  |
| Intercept | -0.58 (-0.94, -0.22)* | -0.42 (-0.86, 0.03) | -0.87 (-1.52 -0.21)* |
| Time | -0.33 (-0.48, -0.18)* | -0.32 (-0.50, -0.13)* | -0.37 (-0.64, -0.10)* |
| Interaction | 0.25 (-0.03, 0.52) | 0.06 (-0.28, 0.40) | 0.63 (0.16, 1.11) * |
| *Negative aspects of the relationship* | |  |  |
| Intercept | 1.02 (0.63, 1.40)* | 1.02 (0.53, 1.52)* | 1.18 (0.54, 1.82)* |
| Time | 0.11 (-0.07, 0.29) | 0.15 (-0.07, 0.37) | 0.04 (-0.26, 0.35) |
| Interaction | -0.27 (-0.60, 0.05) | -0.25 (-0.65, 0.15) | -0.34 (-0.89, 0.21) |
| **From mother** |  |  |  |
| *Emotional support* | |  |  |
| Intercept | -0.66 (-1.65, 0.33) | 0.00 (-1.21, 1.21) | -1.22 (-3.03, 0.58) |
| Time | -0.59 (-0.97, -0.21)* | -0.47 (-0.93, -0.00) | -0.84(-1.52, -0.16)* |
| Interaction | 0.65 (-0.05, 1.35) | 0.57 (-0.28, 1.43) | 0.81 (-0.42, 2.03) |
| *Practical support* | |  |  |
| Intercept | 0.18 (-0.29, 0.66) | 0.20 (-0.39, 0.79) | 0.45 (-0.39, 1.29) |
| Time | -0.39 (-0.57, -0.20)* | -0.33 (-0.56, -0.09)* | -0.51 (-0.82, -0.20)* |
| Interaction | 0.05 (-0.29, 0.39) | 0.02 (-0.41, 0.45) | 0.14 (-0.41, 0.70) |
| *Negative aspects of the relationship* | |  |  |
| Intercept | 0.33 (-0.05, 0.70) | 0.34 (-0.13, 0.81) | 0.48 (-0.17, 1.14) |
| Time | -0.09 (-0.27, 0.09) | -0.07 (-0.29, 0.14) | -0.14 (-0.45, 0.17) |
| Interaction | -0.12 (-0.44, 0.21) | 0.05 (-0.35, 0.44) | -0.37 (-0.92, 0.18) |
| **From mother in law** |  |  |  |
| *Emotional support* | |  |  |
| Intercept | -2.29 (-3.36, -1.23)* | -1.69 (-3.03, -0.36)* | -3.53 (-5.44, -1.62)* |
| Time | -0.89 (-1.26, -0.52)* | -0.99 (-1.42, -0.55) | -0.69 (-1.36, -0.01)* |
| Interaction | 1.54 (0.88, 2.20)* | 1.56 (0.77, 2.36)* | 1.40 (0.20, 2.60)* |
| *Practical support* | |  |  |
| Intercept | -0.56 (-1.04, -0.08)* | -0.48 (-1.07, 0.11) | -0.94 (-1.83, -0.04)* |
| Time | -0.54 (-0.73, -0.36)* | -0.48 (-0.71, -0.25)* | -0.68 (-0.99, -0.36)* |
| Interaction | 0.66 (0.33, 0.99)* | 0.60 (0.19, 1.01)* | 0.80 (0.23, 1.36)* |
| *Negative aspects of the relationship* | |  |  |
| Intercept | 0.61 (0.15, 1.08)* | 0.51 (-0.09, 1.11) | 0.83 (0.06, 1.60) |
| Time | 0.14 (-0.08, 0.36) | 0.18 (-0.10, 0.46) | 0.08 (-0.28, 0.45) |
| Interaction | -0.09 (-0.48, 0.31) | -0.00 (-0.50, 0.49) | -0.24 (-0.88, 0.41) |

| **Supplementary Table S3.** *Mixed model estimations of depression at a given examination point, time and their interaction as predictors of social support over the four examinations* | | | | | |
| --- | --- | --- | --- | --- | --- |
| Nature of support (dependent variable) and predictor | | **B-coefficient (95% CI)** | | | |
|  |  | Unadjusted | Model 1 | Model 2 | Model 3 |
| **From husband** | |  |  |  |  |
| *Emotional support* | | |  |  |  |
| Intercept | -2.64 (-3.49, -1.79)* | | -2.62 (-3.47, -1.76)* | -2.36 (-3.22, -1.50)* | -2.28 (-3.14, -1.42)* |
| Time | -0.07 (-0.44, 0.30) | | -0.07 (-0.44, 0.31) | -0.05 (-0.42, 0.32) | -0.04 (-0.41, 0.33) |
| Interaction | -0.40 (-1.16, 0.36) | | -0.41 (-1.16, 0.35) | -0.44 (-1.19, 0.32) | -0.45 (-1.21, 0.30) |
| *Practical support* | | |  |  |  |
| Intercept | -0.83 (-1.24, -0.42)* | | -0.81 (-1.22, -0.40)* | -0.75 (-1.17, -0.33)* | -0.69 (-0.11, -0.28)* |
| Time | -0.25 (-0.44, -0.07)* | | -0.25 (-0.43, -0.06)* | -0.25 (-0.43, -0.06)* | -0.24 (-0.43, -0.06)* |
| Interaction | 0.05 (-0.32, 0.43) | | 0.05 (-0.32, 0.42) | 0.04 (-0.34, 0.41) | 0.03 (-0.34, 0.41) |
| *Negative aspects of the relationship* | | |  |  |  |
| Intercept | 1.56 (1.11, 2.01)* | | 1.61 (1.66, 2.07)* | 1.50 (1.04, 1.96) * | 1.47 (1.00, 1.94)* |
| Time | 0.16 (-0.05, 0.37) | | 0.17 (-0.05, 0.38) | 0.16 (-0.06, 0.37) | 0.15 (-0.06, 0.37) |
| Interaction | 0.10 (-0.32, 0.52) | | 0.09 (-0.33, 0.51) | 0.12 (-0.30, 0.54) | 0.12 (-0.30, 0.54) |
| **From mother** |  | |  |  |  |
| *Emotional support* | | |  |  |  |
| Intercept | -0.83 (-1.91, 0.25) | | -0.80 (-1.89, 0.28) | -0.73 (-1.82, 0.36) | -0.63 (-1.72, 0.46) |
| Time | 0.13 (-0.34, 0.61) | | 0.13 (-0.35, 0.60) | 0.13 (-0.35, 0.60) | 0.14 (-0.34, 0.61) |
| Interaction | -0.79 (-1.76, 0.18) | | -0.79 (-1.76, 0.18) | -0.76 (-1.73, 0.21) | -0.77 (-1.74, 0.20) |
| *Practical support* | | |  |  |  |
| Intercept | -0.34 (-0.86, 0.19) | | -0.31(-0.84, 0.22) | -0.34 (-0.87, 0.19) | -0.31 (-0.84, 0.22) |
| Time | -0.20 (-0.43, 0.03) | | -0.20 (-0.43, 0.04) | -0.20 (-0.43, 0.03) | -0.20 (-0.43, 0.03) |
| Interaction | -0.13 (-0.61, 0.35) | | -0.13 (-0.61, 0.34) | -0.13 (-0.60, 0.35) | -0.13 (-0.61, 0.35) |
| *Negative aspects of the relationship* | | |  |  |  |
| Intercept | 0.74 (0.28, 1.19)* | | 0.75 (0.30, 1.20)* | 0.73 (0.27, 1.19) * | 0.75 (0.29, 1.21) * |
| Time | 0.09 (-0.12, 0.30) | | 0.10 (-0.11, 0.30) | 0.09 (-0.12, 0.30) | 0.10 (-0.11, 0.31) |
| Interaction | -0.22 (-0.64, 0.20) | | -0.22 (-0.64, 0.20) | -0.21 (-0.63, 0.21) | -0.22 (-0.64, 0.20) |
| **From mother in law** |  | |  |  |  |
| *Emotional support* | | |  |  |  |
| Intercept | -2.23 (-3.22, -1.24)* | | -2.24 (-3.23, -1.25)* | -2.05 (-3.04, -1.06)* | -2.02 (-3.01, -1.03)* |
| Time | -0.50 (-0.94, -0.07)* | | -0.51 (-0.94, -0.07)* | -0.50 (-0.94, -0.07)* | -0.50 (-0.94, -0.07)* |
| Interaction | 0.31 (-0.57, 1.19) | | 0.33 (-0.54, 1.21) | 0.34 (-0.54, 1.21) | 0.35 (-0.52, 1.23) |
| *Practical support* | | |  |  |  |
| Intercept | -1.09 (-1.58, -0.59)* | | -1.07 (-1.57, -0.58)* | -0.99 (-1.49, -0.50)* | -0.97 (-1.47, -0.47)* |
| Time | -0.56 (-0.78, -0.33)* | | -0.56 (-0.78, -0.34)* | -0.57 (-0.79, -0.35)* | -0.57 (-0.79, -0.35)* |
| Interaction | 0.56 (0.12, 1.01)* | | 0.57 (0.12, 1.02)* | 0.59 (0.14, 1.03)* | 0.59 (0.14, 1.03)* |
| *Negative aspects of the relationship* | | |  |  |  |
| Intercept | 0.78 (0.21, 1.34)* | | 0.77 (0.21, 1.34)* | 0.69 (0.11, 1.26)* | 0.66 (0.08, 1.24)* |
| Time | 0.36 (0.09, 0.62) * | | 0.36 (0.09, 0.62) * | 0.33 (0.07, 0.60) * | 0.33 (0.07, 0.59) * |
| Interaction | -0.21 (-0.73, 0.31) | | -0.20 (-0.72, 0.32) | -0.13 (-0.65, 0.39) | -0.12 (-0.64, 0.40) |

Model 1: adjusted for age (categorised into 4 groups: 18-22, 23-25, 26-29, 30+);

Model 2: Model 1 plus education (4 groups: <6, 6-8, 9-11, 12+), number of children (3 groups: 0, 1, 2+), family structure (traditional/nuclear), physical health (3 groups: very good, good and average/poor/very poor), past emotional problems (binary variable);

Model 3: Model 2 plus adverse life events at baseline (4 groups: 0, 1, 2, 3+);

*p<0.05

| **Supplementary Table S4.** *Mixed model estimations of baseline depression, time and their interaction as predictors of social support over the four examinations adjusting postnatal depression* | | | | | |
| --- | --- | --- | --- | --- | --- |
| Nature of support (dependent variable) and predictor | | **B-coefficient (95% CI)** | | | |
|  |  | Unadjusted | Model 1 | Model 2 | Model 3 |
| **From husband** | |  |  |  |  |
| *Emotional support* | | |  |  |  |
| Intercept | -3.62 (-4.81, -2.84)* | | -3.61 (-4.78, -2.44)* | -3.00 (-4.27, -1.73)* | -2.70 (-3.97, -1.43)* |
| Time | -0.35 (-0.73, 0.03) | | -0.35 (-0.73, 0.03) | -0.35 (-0.73, 0.03) | -0.35 (-0.73, 0.03) |
| Interaction | 0.86 (0.17, 1.54)* | | 0.86 (0.18, 1.54)* | 0.86 (0.18, 1.55)* | 0.87 (0.19, 1.55)* |
| *Practical support* | | |  |  |  |
| Intercept | -0.88 (-1.38, -0.37)* | | -0.87 (-1.37, -0.36)* | -0.76 (-1.31, -0.20)* | -0.63 (-1.18, -0.07)* |
| Time | -0.34 (-0.52, -0.15)* | | -0.34 (-0.52, -0.15)* | -0.34 (-0.52, -0.15)* | -0.34 (-0.52, -0.15)* |
| Interaction | 0.41 (0.07, 0.75) * | | 0.41 (0.07, 0.74)* | 0.41 (0.07, 0.74)* | 0.41 (0.08, 0.75)* |
| *Negative aspects of the relationship* | | |  |  |  |
| Intercept | 1.19 (0.67, 1.70)* | | 1.24 (0.73, 1.75)* | 1.12 (0.57, 1.66) * | 1.11 (0.56, 1.66)* |
| Time | 0.27 (0.05, 0.49)* | | 0.27 (0.05, 0.49)* | 0.27 (0.05, 0.49)* | 0.27 (0.05, 0.49)* |
| Interaction | -0.41 (-0.80, -0.02)* | | -0.41 (-0.80, -0.02)* | -0.41 (-0.79, -0.02)* | -0.41 (-0.80, -0.02)* |
| **From mother** |  | |  |  |  |
| *Emotional support* | | |  |  |  |
| Intercept | -1.45 (-2.94, 0.03) | | -1.45 (-2.93, 0.02) | -1.02 (-2.58, 0.54) | -0.69 (-2.24, 0.86) |
| Time | -0.13 (-0.61, 0.34) | | -0.13 (-0.61, 0.34) | -0.14 (-0.62, 0.34) | -0.14 (-0.62, 0.34) |
| Interaction | 0.35 (-0.52, 1.21) | | 0.34 (-0.52, 1.21) | 0.37 (-0.50, 1.24) | 0.38 (-0.48, 1.25) |
| *Practical support* | | |  |  |  |
| Intercept | -0.27 (-0.95, 0.40) | | -0.26 (-0.93, 0.41) | -0.21 (-0.92, 0.49) | -0.12 (-0.83, 0.59) |
| Time | -0.26 (-0.49, -0.02)* | | -0.26 (-0.49, -0.02)* | -0.26 (-0.49, -0.02)* | -0.26 (-0.49, -0.02)* |
| Interaction | 0.12 (-0.30, 0.55) | | 0.12 (-0.31, 0.55) | 0.12 (-0.30, 0.55) | 0.12 (-0.30, 0.55) |
| *Negative aspects of the relationship* | | |  |  |  |
| Intercept | 0.45 (-0.06, 0.96) | | 0.47 (-0.04, 0.98) | 0.46 (-0.08, 1.00) | 0.51 (-0.04, 1.06) |
| Time | 0.11 (-0.10, 0.32) | | 0.11 (-0.10, 0.32) | 0.11 (-0.10, 0.32) | 0.11 (-0.10, 0.32) |
| Interaction | -0.31 (-0.69, 0.08) | | -0.31 (-0.69, 0.08) | -0.30 (-0.69, 0.08) | -0.30 (-0.69, 0.08) |
| **From mother in law** |  | |  |  |  |
| *Emotional support* | | |  |  |  |
| Intercept | -2.07 (-3.64, -0.51)* | | -2.12 (-3.63, -0.61)* | -1.60 (-3.20, 0.00) | -1.46 (-3.07, 0.14) |
| Time | -0.77 (-1.21, -0.34)* | | -0.78 (-1.21, -0.34)* | -0.78 (-1.22, -0.35)* | -0.79 (-1.22, -0.35)* |
| Interaction | 1.37 (0.59, 2.14)* | | 1.38 (0.61, 2.16)* | 1.39 (0.62, 2.17)* | 1.41 (0.63, 2.18)* |
| *Practical support* | | |  |  |  |
| Intercept | -0.25 (-0.97, 0.47) | | -0.27 (-0.97, 0.42) | -0.25 (-0.94, 0.45) | -0.17 (-0.87, 0.53) |
| Time | -0.56 (-0.78, -0.33)* | | -0.56 (-0.78, -0.33)* | -0.57 (-0.79, -0.34)* | -0.57 (-0.79, -0.34)* |
| Interaction | 0.56 (0.16, 0.95)* | | 0.56 (0.17, 0.96)* | 0.56 (0.17, 0.96)* | 0.56 (0.17, 0.96)* |
| *Negative aspects of the relationship* | | |  |  |  |
| Intercept | 1.14 (0.50, 1.77)* | | 1.14 (0.50, 1.78)* | 1.01 (0.33, 1.69)* | 1.00 (0.31, 1.68)* |
| Time | 0.38 (0.11, 0.65)* | | 0.38 (0.11, 0.65)* | 0.37 (0.11, 0.64)* | 0.38 (0.11, 0.64)* |
| Interaction | -0.31 (-0.78, 0.16) | | -0.31 (-0.78, 0.17) | -0.30 (-0.77, 0.17) | -0.30 (-0.77, 0.17) |

Model 1: adjusted for age (categorised into 4 groups: 18-22, 23-25, 26-29, 30+) and postnatal depression at first follow-up;

Model 2: Model 1 plus education (4 groups: <6, 6-8, 9-11, 12+), number of children (3 groups: 0, 1, 2+), family structure (traditional/nuclear), physical health (3 groups: very good, good and average/poor/very poor), past emotional problems (binary variable) and postnatal depression at first follow-up;

Model 3: Model 2 plus adverse life events at baseline (4 groups: 0, 1, 2, 3+) and postnatal depression at first follow-up;

*p<0.05
